# Synthesis, conformational analysis and biological activity of xylopyranosyl sulfur-containing glycosides: dependence of sulfur atom configuration†

**DOI:** 10.1039/d5ra00498e

**Published:** 2025-06-04

**Authors:** Pilar Blasco, Jonas Ståhle, Karin Thorsheim, Axel Furevi, Anna Siegbahn, Emil Tykesson, Gunilla Westergren-Thorsson, Ulf Ellervik, Göran Widmalm

**Affiliations:** a Department of Organic Chemistry, Arrhenius Laboratory, Stockholm University SE-106 91 Stockholm Sweden goran.widmalm@su.se; b Center for Analysis and Synthesis, Center for Chemistry and Chemical Engineering, Lund University P.O. Box 124 SE-221 00 Lund Sweden; c Department of Experimental Medical Science, Lund University, BMC SE-221 00 Lund Sweden

## Abstract

Proteoglycans (PGs) consist of a core protein with covalently bound glycosaminoglycan (GAG) chains that are linked *via* a tetrasaccharide. PGs are important macromolecules that are involved in biological processes such as cell growth and differentiation. A key enzyme in the biosynthesis of PG GAG chains is β-1,4-galactosyltransferase 7 (β4GalT7) that catalyzes the transfer of galactose to a xylose residue in the formation of the linker tetrasaccharide. It is well known that the addition of xylosides containing naphthyl aglycones can initiate the biosynthesis of GAG chains by acting as substrates for β4GalT7. Previous studies have shown that its galactosylation ability is increased by using bioisosters, in which the anomeric oxygen is replaced with sulfur or sulfur-containing functional groups. Thus, 2-naphthyl xylosyl sulfoxides were synthesized and characterized by ^1^H and ^13^C NMR spectroscopy relying on both one- and two-dimensional experiments to differentiate the stereochemistry at the sulfur atom. Notably, the conformationally dependent ^3^*J*_CH_ coupling constants between the anomeric proton and the C2′ atom of the naphthyl group were large and significant, ≥3.3 Hz, for the (*S*)_S_-configured compound as well as for the *O*-glycoside and the thio-derivative whereas the corresponding coupling for the (*R*)_S_-configured compound and the sulfone derivative had ^3^*J*_C2′,H1_ < 0.6 Hz and ^3^*J*_C2′,H1_ < 0.5 Hz, respectively. Quantum mechanical calculations of the ^3^*J*_C2′,H1_ coupling constant corroborated the experimentally observed trends at the *ϕ* torsion angle. The galactosylation by β4GalT7 of the different acceptor substrates showed the highest affinity for the (*R*)_S_-configured compound and the sulfone derivative whereas an intermediate affinity was present for the (*S*)_S_-configured compound and the thio-derivative. The enzyme efficiency exhibited with the latter substrate was more than three times higher than with any other of the thio-derivatives. From molecular docking of the acceptor substrates to the UDP-galactose:β4GalT7 complex specific intermolecular interactions were identified. The binding affinity correlates with stacking to a tyrosine residue and a weak C–H⋯O hydrogen bond between the indole group of tryptophan in the enzyme and a proximate oxygen atom of sulfone and sulfinyl derivatives of 2-naphthyl xylosides.

## Abstract 2

In galactosylation by β4GalT7 of 2-naphthyl xylosyl sulfoxides, higher affinity for the enzyme was observed for the acceptor substrate having the (*R*)_S_-configuration than for the diastereomer with the (*S*)_S_-configuration.

## Graphical abstract

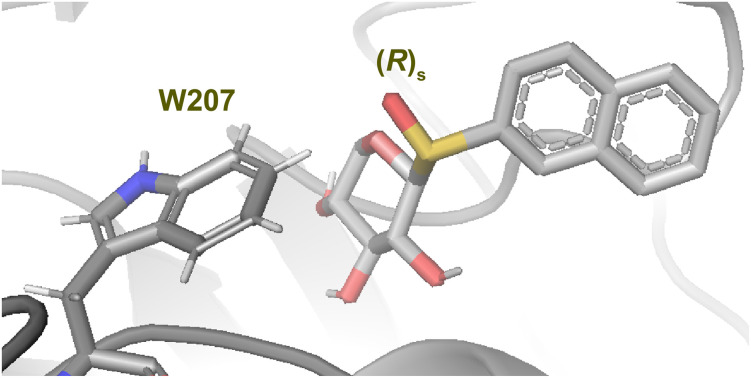

## Introduction

Glycosyltransferases constitute a large family of enzymes that are involved in the biosynthesis of oligosaccharides, polysaccharides and glycoconjugates. These saccharide-containing enzyme products mediate a wide range of functions from structure and storage to signaling. β-1,4-galactosyltransferase 7 (β4GalT7) was identified in 1999, by cloning and expression, as having galactosyltransferase I activity, *i.e.*, catalyzing the transfer of galactose from uridine-5′-diphosphogalactose (UDP-Gal) to a xylose residue in the biosynthesis of proteoglycans (PGs).^1^ Qasba and co-workers later reported the crystal structure of β4GalT7 from both human and *Drosophila*, where a conformational change upon Mn^2+^ and UDP-Gal binding is observed, forming an acceptor binding pocket for the substrate xylose.^2,3^ Crystallization of the ternary enzyme complex showed a Michaelis complex where the coupling of galactose to xylose takes place *via* an S_N_2-type mechanism.^3^ In a previous work, we further investigated the active site of β4GalT7 and observed a very narrow pocket consisting of a precise set of hydrogen bond acceptor residues, where the xylose residue was found to be the ideal substrate.^4^

Proteoglycans (PGs) constitute the majority of the extracellular matrix and they are involved in a variety of biological processes, such as cell growth, brain development, differentiation, and cell adhesion.^5–7^ These macromolecules are important regulators of tumor progression through their interactions with several proteins.^8–13^ These glycoconjugates consist of one or several glycosaminoglycan (GAG) chains that are covalently bound to a core protein *via* a linker tetrasaccharide. The GAG chains are essential for the biological activity of PGs; GAGs interact with growth factors, cytokines, enzymes, and other signaling molecules which are found to be involved in cancer processes.^14,15^ The biosynthesis of PGs is mediated by a cascade of different glycosyltransferases; the process begins with the linking of xylose^16^ to a serine residue of the protein core, followed by the addition of two galactose moieties and one glucuronic acid to form the linker region. This is then elongated by the addition of alternating disaccharide units and modified, through *e.g.* epimerization, *N*-deacetylation, and *O*- and *N*-sulfation, to form different types of GAG chains, *i.e.* heparan sulfate (HS) and chondroitin sulfate/dermatan sulfate (CS/DS).^4,17,18^

It is known that exogenously added xylosides carrying hydrophobic aglycones could induce free GAG chain formation by acting as substrate for β4GalT7. This competition approach was found to cause growth inhibition of tumor cells.^19–23^ Moreover, the modified xylosides could increase the galactosyltransferase activity of the enzyme. Recent results have shown that xylosides carrying aromatic aglycones, *e.g.* a naphthyl moiety, generate efficient β4GalT7 acceptors.^4,19^ Moreover, the positioning of the aromatic moiety and the xylose residue further apart by using a linker was well tolerated by the enzyme without diminishing the activity.^24^ Even more, high bulky aglycones were good acceptors.^25^

**Fig. 1:**
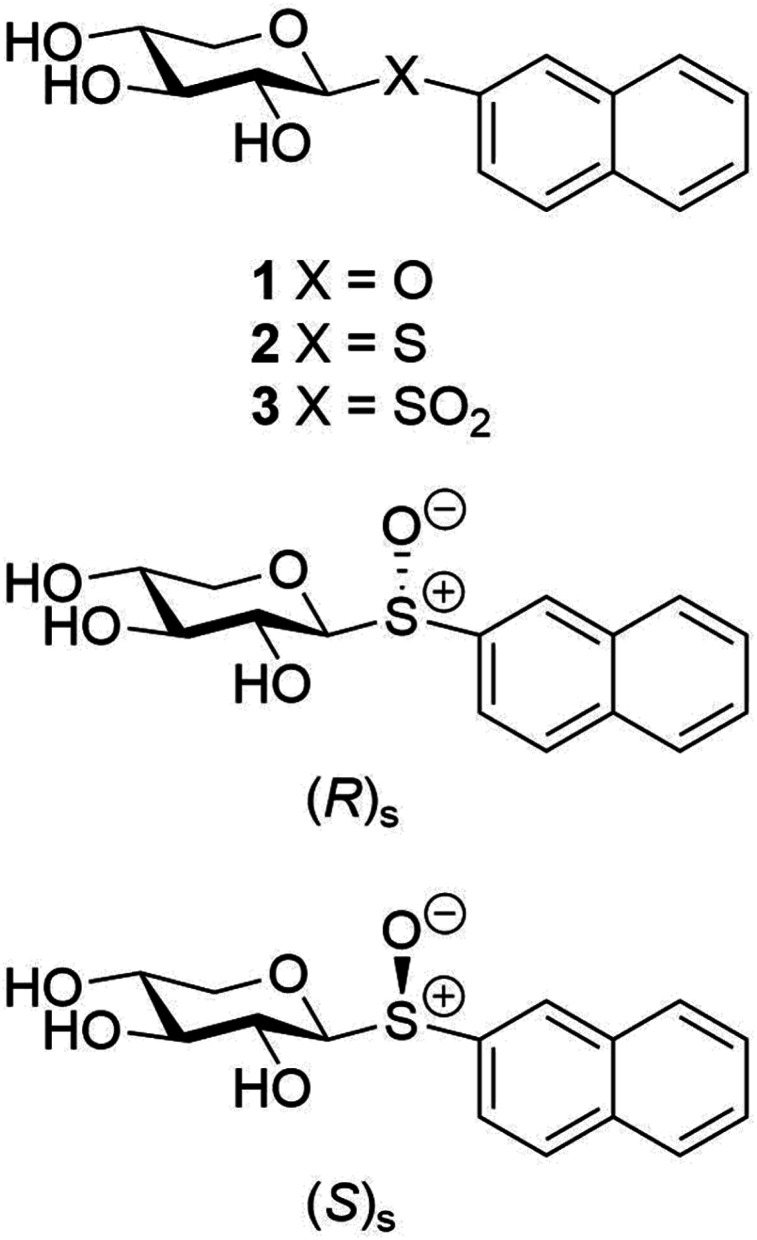
Schematic of 2-naphthyl β-d-xylopyranoside compounds 1–3 and the diastereomeric xylosyl sulfoxides having the (*R*)_S_- and (*S*)_S_-configuration at the sulfur atom.

The anomeric oxygen atom in 2-naphthyl β-d-xylopyranoside (1) can be replaced by a sulfur atom (Fig. 1), generating thioxyloside 2, without losing its activity,^19^ whereas exchanging it to a methylene group, significantly reduced the galactosylation activity.^20^ These results agree with earlier investigations of GAG priming of *S*- and *C*-xylosides.^14,17^ In addition, *N*-xylosides and triazolyl including xylosides have been evaluated in terms of GAG priming ability, where the structure of the aglycone strongly affects their activity.^26–29^

Interestingly, xylosyl sulfone 3, where a sulfonyl group is present at the glycosidic linkage, is several times more efficient in terms of galactosylation compared to xyloside 1 as well as thioxyloside 2.^25^ To further investigate the effect of xyloside modifications, we have synthesized xylosyl sulfoxides (4 and 5) and studied conformational preferences of all the analogues in solution in order to gain insight into their ability to act as substrates for β4GalT7. Sulfoxides possess a stereogenic center at the sulfur atom;^30^ hence diastereomers can be formed with different configuration, (*R*)_S_ and (*S*)_S_, at the sulfinyl sulfur atom (Fig. 1). With these compounds in hand, we investigated the activity of β4GalT7 in galactosylation of xylosyl sulfoxides as acceptors. Moreover, molecular docking of the sulfoxide molecules was also performed to illuminate similarities and differences to 1–3 in the active site of β4GalT7.

## Results and discussion

### Synthesis and diastereomeric differentiation

**Scheme 1:**
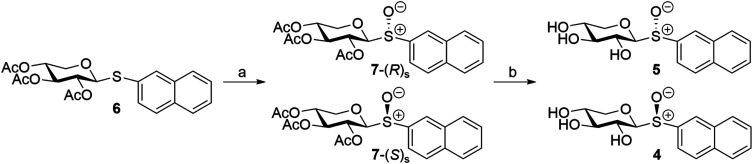
Synthesis of target sulfoxides 4 and 5. Reagents and conditions: (a) *m*CPBA, CH_2_Cl_2_, −78 °C, 2.5 h, 95% as a mixture ((*R*)_S_-7 and (*S*)_S_-7); (b) G/GHNO_3_, MeOH/CH_2_Cl_2_ (9 : 1), rt, 30 min, 64% (4) and 21% (5).

The diastereomeric xylosyl sulfoxides with the (*R*)_S_- and (*S*)_S_-configuration at the sulfur atom (Fig. 1) were synthesized by a two-step procedure from thioxylopyranoside 6 by oxidation using *m*CPBA at −78 °C, which generated sulfoxides 7-(*R*)_S_ and 7-(*S*)_S_ in an excellent 95% yield, but as an inseparable mixture (Scheme 1). The relative ratio between the major and the minor product was 7 : 3 based on ^1^H NMR analysis of the mixture. A diastereomeric product was anticipated since equatorial thioglycosides are known to show poor selectivity compared to the axial analogues in the oxidation reaction forming sulfoxides.^31,32^ This may be explained by conformational preferences due to the *exo*-anomeric effect where predominantly the *S*_S_ configuration is obtained when oxidizing equatorial thioglycosides and *R*_S_ is almost exclusively generated when oxidizing a thioglycoside with an axially oriented C–S bond.

For equatorial glycosyl *S*_S_ sulfoxides the ^13^C NMR chemical shift of C1 is observed more downfield than the analogous *R*_S_ sulfoxides.^33,34^ In the product mixture of 7-(*R*)_S_ and 7-(*S*)_S_ the major diastereomer has for its anomeric carbon an NMR chemical shift of 93.4 ppm whereas the minor isomer shows the corresponding chemical shift upfield at 91.8 ppm, *i.e.* Δ*δ*_C_^*R*–*S*^ −1.6, which, according to literature data of similar compounds, makes it possible to assign the major isomer of the xylosyl sulfoxide to the (*S*)_S_-configuration and, consequently, the minor isomer to the (*R*)_S_-configuration. Furthermore, the two diastereomers of phenyl 2,3,4,6-*tetra-O*-benzoyl-1-sulfinyl-β-d-glucopyranoside differ, *inter alia*, in the ^1^H NMR chemical shift of their anomeric protons,^35^ being 4.87 ppm and 4.62 ppm for the (*S*)_S_- and (*R*)_S_-isomers, respectively, *i.e.* Δ*δ*_H_^*R*–*S*^ −0.25. Likewise, the ^1^H NMR chemical shift of H1 for the herein per-*O*-acetylated 2-naphthyl derivative of the major isomer is observed at 4.57 ppm whereas that of the minor isomer resonates at 4.32 ppm, *i.e.* Δ*δ*_H_^*R*–*S*^ −0.25, consistent with the conclusion made based on ^13^C NMR data.

De-*O*-acetylation of the mixture of 7-(*R*)_S_ and 7-(*S*)_S_ using standard Zemplén conditions generated partial cleavage of the glycosidic linkage forming methyl β-d-xylopyranoside, which was difficult to separate from the desired target sulfoxides. However, when using a solution of guanidine/guanidinium nitrate^36^ the hydrolysis of the glycosidic bond could be prevented and both diastereomers were separated by column chromatography. The major compound (4) was eluted first followed by the minor compound (5). Based on the fact that *m*CPBA oxidation of phenyl 2,3,4-tri-*O*-benzoyl-1-thio-β-d-xylopyranoside gives a 7 : 3 ratio of the (*S*)_S_- and (*R*)_S_-configuration at the sulfur atom, we tentatively assign compound 4 as having the former and compound 5 as having the latter stereochemistry (Scheme 1).^37^ In addition, the UV spectra of compounds 4 and 5 were closely similar and showed absorption band maxima at ∼225 nm while the CD spectra at this wavelength were of opposite signs, consistent with different chirality at the sulfur atom^38,39^ in the two compounds.

### Xyloside conformation by NMR and QM

#### NMR chemical shift comparison

1D ^1^H and ^13^C NMR experiments in combination with 2D ^1^H,^1^H- and ^1^H,^13^C-correlated experiments were used to corroborate and assign ^1^H and ^13^C NMR chemical shifts of compounds 1–5 in methanol-*d*_4_. The ^1^H chemical shifts were subsequently refined aided by NMR spin-simulation of spectra based on a total line-shape analysis.^40^ However, a residual pentet CHD_2_OD peak from the solvent overlapped with a multiplet resonances in 4, which interfered with the total line-shape analysis. This was resolved by a WEFT NMR experiment^41^ in which the solvent peak was removed from the spectrum by choosing a suitable delay time of the inversion-recovery pulse sequence that is the basis for the solvent suppression experiment. The chemical shift difference for the anomeric carbon in 4*vs.*5 is smaller, 0.5 ppm, than that observed for the protected compounds (*vide supra*). For the major compound 4 the C1 chemical shift is higher than for the minor compound 5, like for 7-(*S*)_S_*vs.*7-(*R*)_S_, indicating that 4 has the (*S*)_S_-configuration and that 5 has the (*R*)_S_-configuration; consequently Δ*δ*_C_^5–4^ −0.5. Likewise, for the anomeric protons, Δ*δ*_H_^5–4^ −0.34. However, for the H2 proton the chemical shift displacement is reversed^32^ and larger,Δ*δ*_H_^5–4^ 0.42, further corroborating the assigned stereochemistry.

#### NMR and QM based conformational analysis

**Fig. 2:**
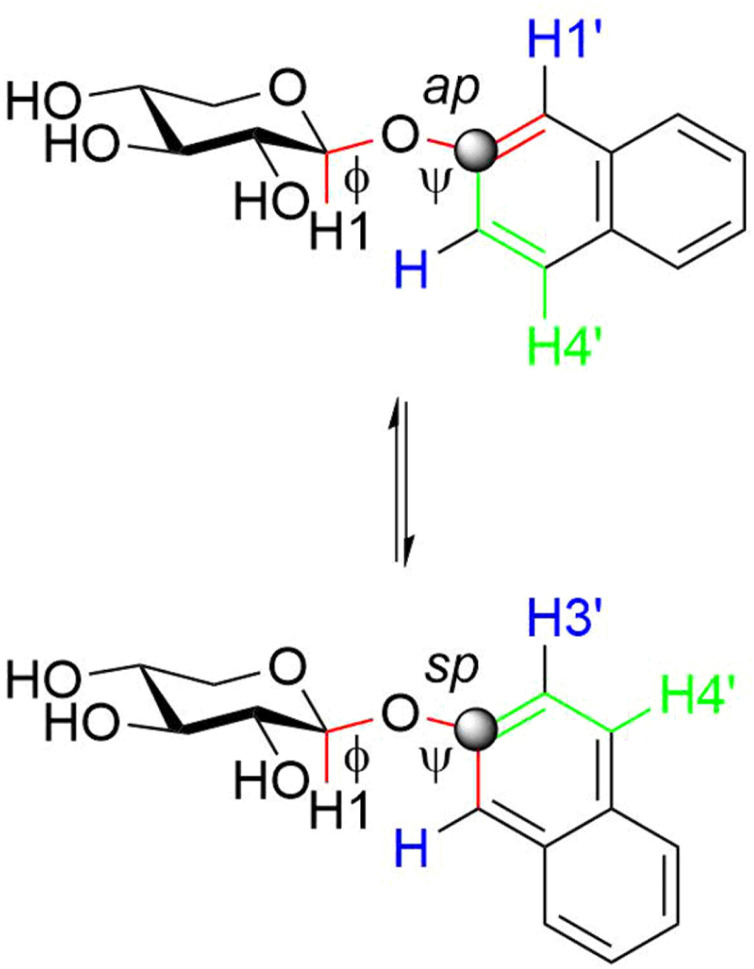
Schematic of compound 1 illustrating ^3^*J*_CH_ coupling pathways from C2′ to H1 and to H4′. In 1D ^1^H,^1^H-NOESY experiments irradiation at the H1 resonance frequency results in NOE peaks due to cross-relaxation to, *inter alia*, H1′ and H3′ (the latter two atoms are shown in blue color).

**Fig. 3:**
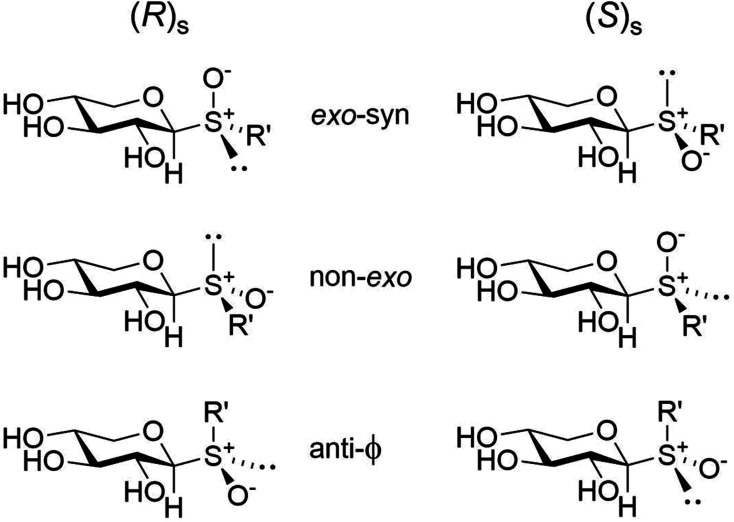
Schematic of the three rotamers at the glycosidic *ϕ* torsion angle for the diastereomeric xylosyl sulfoxides and pertinent description of their conformational states.

NMR experiments suitable for addressing conformational preferences at the glycosidic linkage of the 2-naphthyl xylosyl glycosides include, *inter alia*, 1D ^1^H,^1^H-NOESY^42^ and heteronuclear one-dimensional long-range (1DLR)^43,44^ techniques. In the former experiment correlations from the anomeric proton to H1′ and/or H3′ in the naphthyl group can be anticipated being dependent on the conformation(s) at the glycosidic linkage; intraresidue NOEs from H1 to H3 or alternatively to H5_pro-S_ may be used (Fig. 2), in conjunction with a molecular model, to obtain a proton–proton reference distance *r*_ref_ for determination of an effective distance *r*_*ij*_ across the glycosidic linkage using the isolated spin-pair approximation (ISPA)^45^ and the relationship *r*_*ij*_ = *r*_ref_(*σ*_ref_/*σ*_*ij*_)^1/6^ where *r*_ref_ is the known reference distance from the molecular model, *σ*_ref_ and *σ*_*ij*_ are the cross-relaxation rates for the reference interaction and the interaction between spins *i* and *j*, respectively. This reasoning is based on the fact that the β-d-xylopyranosyl residue is present in the ^4^*C*_1_ chair conformation.^46,47^ In the latter technique the ^1^H,^13^C-heteronuclear spin–spin coupling constant related to the torsion angle *ϕ* (Fig. 2) is to be extracted. The torsion angle dependence can be described by Karplus-type relationships^48,49^ for ^3^*J*_CH_ where the atom-sequences C–O–C–H and C–S–C–H are present in compounds 1 and 2, respectively. To proceed with the conformational analysis quantum mechanics (QM) geometry optimized models were computed, which for *ϕ* are referred to as *exo-syn*, non-*exo* and *anti-ϕ*,^50^ exemplified for the (*R*)_S_ and (*S*)_S_ sulfoxide isomers (Fig. 3), whereas for the torsion angle *ψ* (Fig. 2) defined by the atom sequence C1–X–C2′–C1′ where X = O, S the conformational states are referred to as *syn*-periplanar (sp) for which *ψ* ≈ 0° and anti-periplanar (ap) where *ψ* ≈ 180°. 1D ^1^H,^1^H-NOESY buildup curves for compounds 1–5 were obtained by selective excitation of the anomeric proton and cross-relaxation rates were extracted using the PANIC approach;^51^ subsequent analysis using ISPA and quantum mechanical (QM) geometry optimized structures resulted in effective proton–proton distances (Table 1).

**Table 1:** Proton–proton cross-relaxation rates (*σ*_*ij*_), experimentally derived distances (*r*_NMR_) and reference distances (*r*_ref_) from QM geometry optimized structures with the xylopyranoside in the ^4^*C*_1_ chair conformation for compounds 1–5 together with their glycosidic torsion angles

Proton pair		Compound				
		1	2	3	4	5
H1,H3	*σ*_NMR_/s^−1^		0.0298		0.0296	0.0345
	*r*_ref_/Å		2.62		2.64	2.70
H1–H5_pro*-S*_	*σ*_NMR_/s^−1^	0.0295		0.0376		
	*r*_ref_/Å	2.44 (2.49)^a^		2.53		
H1,H1′	*σ*_NMR_/s^−1^	0.0434	0.0100	0.0071	0.0048	0.0093
	*r*_NMR_/Å	2.29 (2.21)^a^	3.14 (2.24)^b^	3.34	3.58	3.36
H1,H3′	*σ*_NMR_/s^−1^	0.0110	0.0076	0.0086	0.0087	0.0194
	*r*_NMR_/Å	2.88 (4.32)^a^	3.29 (4.76)^b^	3.24	3.24	2.97

**Torsion angle**						
*ϕ*	H1–C1–X–C2′/°	42 (46)^a^	33	50	34	–63
*ψ*	C1–X–C2′–C1′/°	17 (1)^a^	40	96	87	–107

The glycosidic torsion angles of the geometry optimized compound 1 are quite similar to that of the crystal structure^52^ of the compound (Table 1), *i.e.*, *ϕ* has the *exo*-anomeric conformation and *ψ* an sp conformation. In this arrangement the solid state structure shows a short H1, H1′ distance of 2.2 Å whereas a long H1, H3′ distance of 4.3 Å is observed. In solution the population averaged distance of the former is slightly longer, 2.3 Å, whereas the latter is notably decreased to 2.9 Å. By noting that the barrier to rotation for an aryl methoxy group such as in anisole is low, on the order of 3 kcal mol^−1^,^53^ and that the barrier to rotation of the naphthyl group at the *ψ* torsion angle has been estimated to be of similar magnitude^47^ the rotation of the aglycone at *ψ* is considered to be unrestricted, but the conformational preference of the *ψ* torsion is still favoring an sp conformation, since the distance H1, H1′ < H1, H3′. In the thioxyloside 2 and the sulfone derivative 3 the population-averaged distance from the anomeric proton to those adjacent to the linkage position C2′ are similar, *i.e.*, H1, H1′ ≈ H1, H3′ supporting unrestricted rotation and similar populations of sp and ap conformations at the *ψ* torsion angle. The (*S*)_S_- and (*R*)_S_-sulfinyl derivatives 4 and 5, respectively, contrast the conformational preference in 1; both have the distance for H1, H1′ > H1, H3′ indicating a small preference for the ap conformation at *ψ*.

**Fig. 4:**
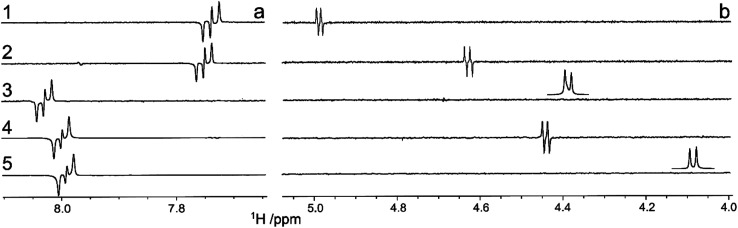
^1^H-detected spectra from 1D long-range experiments of compounds 1–5 where upon selective excitation at the C2′ resonance frequency heteronuclear correlations are observed to (a) H4′ in all compounds and (b) to H1 in compounds 1, 2 and 4. Inserts show the anomeric proton resonance for 3 and 5 at their respective chemical shift, since for these compounds the long-range correlations were not detected; consequently it was concluded that ^3^*J*_C2′,H1_ < 0.5 Hz and ^3^*J*_C2′,H1_ < 0.6 Hz, respectively.

^1^H,^13^C-Heteronuclear coupling constants were obtained by the 1DLR experiment. In the rigid planar naphthyl group ^3^*J*_C2′,H4′_ coupling constants are large (Fig. 4a) and of similar magnitude in all five compounds (Table 2). The ^3^*J*_C2′,H1_ in compound 1, as seen from the anti-phase peak separation in the NMR spectrum (Fig. 4b), is for a transglycosidic heteronuclear coupling constant of intermediate magnitude (Table 2) and the corresponding one in 2 is similar to that of 2-naphthyl 1,5-dithio-β-d-xylopyranoside, with a sulfur atom at the glycosidic linkage, being 4.1 Hz.^52^ However, peaks from scalar coupled C2′ and H1 nuclei are absent for the sulfone-containing compound 3 as well as for the (*R*)_S_-sulfoxide derivative 5, whereas in compound 4 having the (*S*)_S_-chirality at the sulfur atom the magnitude of the coupling constant is similar to those of 1 and 2 (Fig. 4b and Table 2). The absence of detectable ^3^*J*_C2′,H1_ couplings in 3 and 5 makes it possible to determine an upper limit of these coupling constants. The natural line-width of these two samples under the experimental conditions employed was *ν*_1/2_ ≈ 1.0 Hz. For an anti-phase peak a limiting separation is reached at ∼0.576 times the line-width beyond which the only effect of a smaller *J* coupling is to reduce the overall intensity of the anti-phase peak.^54^ Thus, in compounds 3 and 5 the scalar interaction ^3^*J*_C2′,H1_ < |0.5| Hz and ^3^*J*_C2′,H1_ < |0.6| Hz, respectively (Table 2).

**Table 2:** Experimental NMR heteronuclear ^3^*J*_CH_ (in Hz, absolute values) from 1DLR spectra and calculated using QM geometry optimized structures (^4^*C*_1_) for compounds 1–5

Atom pair		Compound				
		1^a^	2	3	4	5
C2′,H4′	Expt	10.7	10.1	9.8	9.7	9.8
	Calc	11.2	10.8	10.9	10.2	10.1
C2′,H1	Expt	3.6	4.2	<0.5	3.3	<0.6
	Calc	3.4	4.3	−0.8	3.4	0.4

a ^3^ *J*_C2′,H1_ = 3.3 Hz from IPAP-selHSQMBC NMR experiments.

**Fig. 5:**
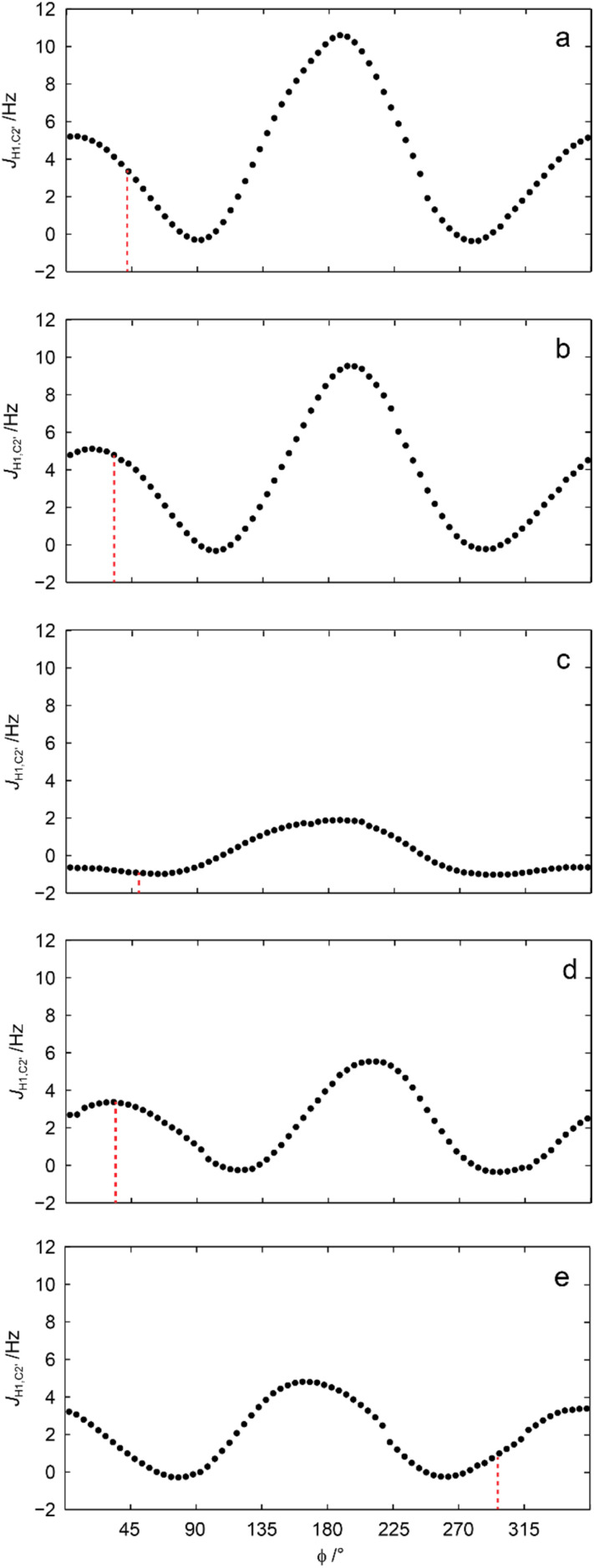
Calculated heteronuclear ^3^*J*_C2′,H1_*vs.* the glycosidic *ϕ* torsion angle for compounds 1–5 (a–e, respectively); the vertical dashed lines correspond to the *ϕ* torsion angle of the geometry optimized structures described in Table 1. A conductor-like polarizable continuum model was used for methanol as solvent.^69,70^

This conspicuous observation was further investigated by QM-based calculations of ^3^*J*_C2′,H1_ as a function of the torsion angle *ϕ*. The ^3^*J*_C2′,H4′_ coupling constants in geometry optimized 1–5 were well reproduced at the level of theory used and so were ^3^*J*_C2′,H1_ related to *ϕ* (Table 2). Subsequent scanning of the *ϕ* torsion angle in 5° increments and geometry optimization at each torsion angle followed by calculation of ^3^*J*_C2′,H1_ resulted in computed values (Fig. 5) unveiling its conformational dependence, especially, that ^3^*J*_C2′,H1_ is small for all conformations related to the torsion angle *ϕ* in the sulfone derivative, *i.e.*, compound 3 (Fig. 5c).

### β4GalT7 assay

A truncated version of β4GalT7 fused with glutathione *S*-transferase was expressed in *E. coli* and used in an enzymatic assay to measure the galactosylation by β4GalT7.^19^ Various concentrations of compounds 1–5 were incubated with the recombinant β4GalT7 enzyme and the donor substrate, UDP-Gal, for 30 min at 37 °C. The reaction progress was analyzed using HPLC with fluorescence detection and the identities of the galactosylated products were corroborated by LCMS analysis (Table S1†). The kinetic parameters were calculated using nonlinear regression to the Michaelis–Menten model (Table 3).

**Table 3:** Galactosylation of xylosides by β4GalT7

Compound	*K*_m_/mM	*V*_max_/pmol s^−1^	*k*_cat_/s^−1^	*k*_cat_/*K*_m_/mol^−1^ s^−1^
1^a^	0.70	1.6	1.2	1.6
2^a^^,^^b^	0.30	1.7	1.8	5.8
3^b^	0.10	2.1	2.2	21
4 (*S*)_S_	0.37	2.5	2.5	6.9
5 (*R*)_S_	0.14	1.1	1.1	7.8

a Slightly different kinetic parameters for 1 and 2 are obtained compared to previously published data since different batches of enzymes were used, which affect the parameters slightly.

b Kinetic parameters were obtained for concentrations up to the highest observed reaction rate.

Between all substrates, the highest affinity was observed for analogs 3 and 5; compounds 2 and 4 showed an intermediate affinity, while the oxygen-containing compound 1 showed the lowest affinity, as measured by the *K*_m_ values. Interestingly, the (*S*)_S_-configuration sulfoxide 4 showed the highest turnover, as indicated by the *k*_cat_, whereas the (*R*)_S_-sulfoxide 5 showed the lowest. Analog 1 showed similar *k*_cat_ as 5.

Moreover, the highest enzyme efficiency (*k*_cat_/*K*_m_) was observed for sulfone 3 whereas the sulfoxides 4 and 5 showed very similar values. The lowest efficiency was present towards 1. In the case of analog 4 ((*S*)_S_-chirality) the turnover (*k*_cat_) was high whereas for analog 5 having the (*R*)_S_ configuration the turnover was low, but the affinity high. The combined and potentially additive effects may thus result in stereochemical arrangements for donor molecule 3 in the active site of the enzyme thereby favoring the high efficiency.

### Molecular docking of xylosyl acceptors

Molecular docking was used to obtain further insights on atomic interactions in the binding pocket of β4GalT7 and to shed light on the results of the biochemical assay. The protein crystal structures of *Drosophila* β-1,4-galactosyltransferase 7 mutant D211N (PDB ID: 4M4K and 4LW3) devoid of water molecules and xylose-containing acceptors but containing manganese and UDP-galactose were used for the computational studies as well as the enzyme containing the catalytic base Asp211 instead of the mutant having Asn211. The previous molecular docking procedures resulted in identification of hydrogen bonding networks between the amino acids of the enzyme and the three hydroxyl groups in the xylose moiety, which was oriented above Tyr177, whereas the naphthyl aglycone adopted a face-to-face parallel stacking to Tyr179, and consistent with a product-forming nucleophilic attack from the acceptor molecule on the anomeric carbon of the nucleotide sugar the O4 atom of the xylose moiety was in proximity to the C1 atom of UDP-galactose.^4,19,52^ In the present molecular modeling approach the number of sidechains treated as flexible was increased to encompass eight amino acids, *viz.*, K176, Y177, H178, Y179, L209, E210, N211/D211 and D212.

From the molecular docking of compounds 1–5 to the galactosyl transferase based on two crystal structures containing the donor UDP-galactose in the active site the binding affinities were calculated for the highest ranked poses for the mutant containing N211 and the wild type having instead the D211 residue (Table 4). Irrespective of the mutated amino acid 211 or the crystal structures the binding affinities showed a consistent pattern with the lowest affinity for XylONap (1), followed by an increased affinity for XylSNap (2), and further increased closely similar affinities for the sulfone XylSO2Nap (3), sulfoxide XylSOSNap (4), and XylSORNap (5) derivatives.

**Table 4:** Binding affinities (kcal mol^−1^) of the highest ranked pose of compounds 1–5 docked using Auto-dock Vina to protein structures containing manganese and UDP-galactose based on the crystal structures from PDB entries 4M4K and 4LW3. The sidechains of K176, Y177, H178, Y179, L209, E210, N211/D211 and D212 were chosen as being flexible

	Ligand	4M4K-N211	4M4K-D211	4LW3-N211	4LW3-D211
1	XylONap	−7.9	−8.0	−7.9	−8.0
2	XylSNap	−8.3	−8.2	−8.3	−8.3
3	XylSO2Nap	−8.5	−8.6	−8.5	−8.5
4	XylSOSNap	−8.5	−8.5	−8.5	−8.5
5	XylSORNap	−8.5	−8.6	−8.5	−8.5

**Fig. 6:**
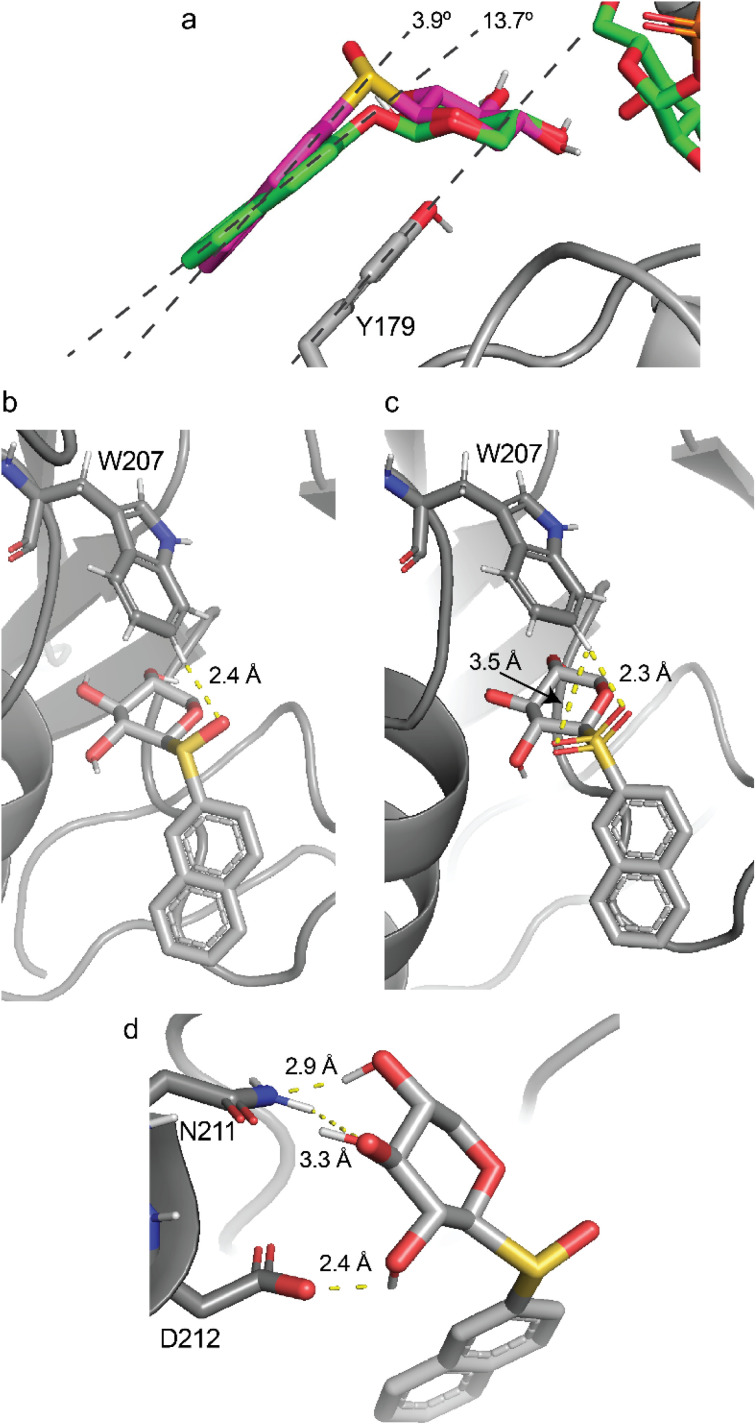
Molecular docking complex of β4GalT7 mutant 4LW3 N211 (a) XylONap (1) stacked to Y179, (b) (*R*)_S_-sulfoxide derivative 5 interaction with W207, (c) the corresponding (*R*)_S_ oxygen atom in the sulfone-containing compound 3 interaction with W207. Molecular docking complex of β4GalT7 mutant 4M4K N211, (d) compound 5 N211 interacts with O2 and O3 of the xylosyl residue and D212 interacts with O4 of the xylosyl residue.

The valence angle C1–O–C2′ at the glycosidic linkage is ∼119° in XylONap (1), whereas for the sulfur-containing compounds it is lower, *viz.*, in XylSNap (2) the C1–O–C2′ angle is ∼103°, in XylSO2Nap (3) it is ∼106°, and in 4 and 5 the valence angle is ∼99°. The aglycon stacking to Y179 was larger for 1 with an angle in the range 8–14° compared to 1–7° in 2–5 for the four different systems, (*i.e.*, amino acid residues D211 and N211 in the protein and crystal structures 4LW3 and 4M4K), consistent with a more favorable stacking interaction (Fig. 6a) and higher binding affinity (Table 4). These results are in line with the *K*_m_ values measured, being highest for 1. Atom–atom interactions that differ between docked complexes are those between the hydrogen atom on the C6 carbon of the indole group of tryptophan 207 (W207). In the 4LW3-based complexes with either D211 or N211 the distance to the glycosidic oxygen in 1 is long being 3.6 Å, whereas it is shorter to the glycosidic sulfur atom in 2 being 2.9 Å, as well as to the oxygen atom at the glycosidic linkage of the (*S*)_S_-sulfinyl derivative 4 being 3.2–3.3 Å. Notably, the shortest distance of 2.4 Å is observed for the oxygen atom of (*R*)_S_-sulfoxide derivative 5 (Fig. 6b) and the corresponding (*R*)_S_ oxygen atom in the sulfone-containing compound 3, being 2.3 Å (Fig. 6c) and 2.4 Å in the D211 or N211 complexes, respectively. These short distances in 3 and 5 are contrasted with long distances between the hydrogen atom on the C6 carbon of the oxygen atom at the glycosidic linkage of the (*S*)_S_-sulfinyl derivative 4 and the corresponding (*S*)_S_ oxygen atom in the sulfone-containing compound 3, in a range of 3.2–3.5 Å. The corresponding distances in the 4M4K-based complexes were similar or slightly longer by a few tens of an Ångström. Thus, the interatomic distances in the docked complexes of 3.6, ∼3.0 and 2.4 Å correlate with the *K*_m_ values of 0.70 (observed for 1), ∼0.35 (2 and 4) and ∼0.10 mM (3 and 5) respectively, suggesting that an additional atom–atom interaction may be of importance in determining the binding affinity. These findings are consistent with the presence of a weak C–H⋯O hydrogen bond,^55,56^ with a favorable interaction energy of <1 kcal mol^−1^ between W207 and a suitably oriented oxygen atom of the ligand. The less favorable interactions for 1 may consequently lead to a lower efficiency of the enzyme with the glycosidic oxygen-containing compound as measured by *k*_cat_*/K*_m_ (Table 3). We also illustrate interatomic interactions in one of the complexes (N211/4M4K) where for compound 5 D212 interacts with O2 of the xylosyl residue and N211 interacts with O3 and O4 of the xylosyl residue, with short distances of 2.4, 3.3 and 2.9 Å, respectively (Fig. 6d), similar to those observed in the crystal structure being 2.7, 3.2 and 2.7 Å, respectively.^3^

## Conclusions

The diastereomeric pair of 2-naphthyl β-d-xylopyranoside sulfoxides were synthesized, purified and characterized, in particular by NMR spectroscopy and quantum mechanical calculations, identifying them as having the (*R*)_S_- and (*S*)_S_-configuration at the sulfur atom for compounds 5 and 4, respectively. Transglycosidic coupling constants at *ϕ* in oligosaccharides often show ^3^*J*_CH_ ≈ 4 Hz^57^ as a result of having an *exo*-anomeric conformation at this torsion angle, also observed for the *O*- and *thio*-glycosides 1 and 2, respectively, as well as for the (*S*)_S_-configured sulfoxide 4. The glycosidic torsion angles in these QM geometry optimized structures have *ϕ* ≈ 40° and computed coupling constants revealed ^3^*J*_C2′,H1_ ≈ 4 Hz for the conformers. However, the magnitude of ^3^*J*_C2′,H1_ was small for all conformations of the sulfone derivative (3), and not detectable by the 1DLR NMR experiment, even though the glycosidic conformation at *ϕ* = 50° of the low-energy conformer. Furthermore, the ^3^*J*_C2′,H1_ coupling was not detectable in (*R*)_S_-configured sulfoxide 5, for which the low-energy conformer had a *gauche*^−^ conformation at the *ϕ* torsion angle. The importance of stereoelectronic effects in sulfide-, sulfoxide- and sulfone-containing compounds^58,59^ on conformational preferences and the observed differences in ^3^*J*_CH_ coupling constants on these glycosides and similar compounds await further detailed studies to clarify the relative importance of steric and electronic effects.

The activity of β4GalT7 in galactosylation reactions was analyzed and the reaction progress was monitored by identification of the products containing a galactosyl residue. The highest affinity was observed for the (*R*)_S_-configured compound and the sulfone derivative and the highest enzyme efficiency (*k*_cat_/*K*_m_) was observed for sulfone-containing compound. Molecular docking of the acceptor substrates to the UDP-galactose:β4GalT7 complex identified aglycon stacking to Y179 as being more favorable in the derivatives of the 2-naphthyl xylosides than in the parent compound. Furthermore, the binding affinity correlated with the stacking to the tyrosine residue and a weak C–H⋯O hydrogen bond between the indole group of a tryptophan residue. To elucidate if an oxygen atom of sulfone and sulfinyl derivatives of these 2-naphthyl xylosides is geometrically arranged to participate in a C–H⋯O hydrogen bond to the enzyme, X-ray crystallography studies of these derivatives and β4GalT7 would be of great interest to perform in future studies, thereby facilitating additional specific information on protein:ligand complexes in β4GalT7,^60,61^ as a stepping-stone to further studies on β-1,4-GalT from bovine milk^62^ or on the human galactosyltransferase β3GalT5.^63^

## Materials and methods

### Synthesis

All moisture- and air-sensitive reactions were carried out under an atmosphere of dry nitrogen using oven-dried glassware. Solvents were dried prior to use. Purchased reagents were used without further purification. Organic phases were dried using Biotage ISOLUTE Phase separators. Chromatographic separations were performed on a Biotage Isolera One flash purification system using Biotage SNAP KP-Sil silica cartridges. Thin-layer chromatography was performed on precoated TLC alumina plates coated with silica gel 60 F_254_ 0.25 mm (Merck). Spots were visualized with UV light or by staining with *para*-anisaldehyde. NMR spectra of synthetic intermediates were recorded at room temperature using a Bruker Avance II 400 MHz spectrometer in CDCl_3_ from which chemical shifts (in ppm) were referenced to residual *δ*_H_ 7.26 and to *δ*_C_ 77.16. Mass spectra were recorded on Micromass Q-TOF micro™. Synthesis and physical characterization of compounds 1,^46^2 (ref. 19) and 3 (ref. 19) have been previously reported.

### 2-Naphthyl 1-thio-β-d-xylopyranoside (*S*)_S_-oxide 4 and 2-naphthyl 1-thio-β-d-xylopyranoside (*R*)_S_-oxide 5

2-Naphthyl 2,3,4-tri-*O*-acetyl-1-thio-β-d-xylopyranoside (6) (307 mg, 0.734 mmol) was dissolved in CH_2_Cl_2_ (6 mL) and cooled to −78 °C. *m*CPBA (180 mg, 0.78 mmol) dissolved in CH_2_Cl_2_ (3 mL) was added dropwise and after 2.5 h, the reaction mixture was allowed to reach rt. Satd aq NaHCO_3_ (10 mL) was added and the organic phase was dried before concentrated under reduced pressure. Column chromatography (SiO_2_, 25 → 50% EtOAc in heptane) gave a mixture of the diastereomers (*R*)_S_-7 and (*S*)_S_-7. The ratio between the major and the minor isomer was 7 : 3, based on the ^1^H NMR spectrum. Evaporation of the solvent furnished the products as a white solid (304 mg, 95%). HRMS calcd for C_21_H_22_O_8_SNa [M + Na]^+^: 457.0933; found: 457.0930. Major isomer (relative intensity 0.70): ^1^H NMR: *δ* 8.18 (1H, H-1′), 7.97–7.90 (m, 3H, H-4′, H-5′, H-7′), 7.64 (dd, *J*_H3′,H4′_ 8.64, *J*_H3′,H1′_ 1.78, 1H, H-3′), 7.59–7.58 (m, 2H, H-8′, H-6′), 5.34 (dd, *J*_H1,H2_ 8.26, *J*_H2,H3_ 8.04, 1H, H-2), 5.24 (dd, *J*_H2,H3_ 8.04, *J*_H3,H4_ 8.35, 1H, H-3), 4.85 (ddd, *J*_H3,H4_ 8.35, *J*_H4,H5eq_ 4.89, *J*_H4,H5ax_ 8.49, 1H, H-4), 4.57 (d, *J*_H1,H2_ 8.26, 1H, H-1), 4.25 (dd, *J*_H4,H5eq_ 4.89, *J*_H5ax,H5eq_ −11.76, 1H, H-5_eq_), 3.48 (dd, *J*_H5ax,H4_ 8.49, *J*_H5ax,H5eq_ −11.76, 1H, H-5_ax_), 2.04, 2.00, 1.98 (3 s, 9H, CH_3_). ^13^C NMR: *δ* 170.00, 169.75, 169.28 (3 CO), 136.64, 134.79, 132.76 (3 Ar), 129.22 (C-4′), 128.76 (C-7′), 128.22 (C-8′), 128.11 (C-5′), 127.45 (C-6′), 126.22 (C-1′), 93.43 (C-1), 71.79 (C-3), 67.96 (C-4), 66.50 (C-5), 66.29 (C-2), 20.75, 20.74, 20.51 (3 Me). Minor isomer (relative intensity 0.30): ^1^H NMR: *δ* 8.18 (1H, H-1′), 7.94–7.90 (m, 3H, H-4′, H-5′, H-7′), 7.60–7.58 (m, 3H, H-3′, H-6′, H-8′), 5.37 (dd, *J*_H2,H1_ 9.24, *J*_H2,H3_ 8.84, 1H, H-2), 5.26 (dd, *J*_H3,H2_ 8.84, *J*_H3,H4_ 8.87, 1H, H-3), 4.98 (ddd, *J*_H4,H3_ 8.87, *J*_H4,H5eq_ 5.26, *J*_H4,H5ax_ 9.26, 1H, H-4), 4.32 (d, *J*_H1,H2_ 9.24, 1H, H-1), 4.19 (dd, *J*_H5eq,H4_ 5.26, *J*_H5eq,H5ax_ −11.58, 1H, H-5_eq_), 3.28 (dd, *J*_H5ax,H4_ 9.26, *J*_H5ax,H5eq_ −11.58, 1H, H-5_ax_), 2.04, 2.00, 1.98 (3 s, 9H, CH_3_). ^13^C NMR: *δ* 170.31, 169.63, 169.12 (3 CO), 136.20, 134.84, 132.83 (3 Ar), 129.35 (C-4′), 128.72 (C-7′), 128.19 (C-5′), 128.15 (C-8′), 127.45 (C-6′), 126.54 (C-1′), 120.77 (C-3′), 91.78 (C-1), 72.85 (C-3), 68.34 (C-4), 67.35 (C-2), 67.11 (C-5), 20.79, 20.71, 20.66 (3 Me).

The mixture of compounds (*R*)_S_-7 and (*S*)_S_-7 (152 mg, 0.349 mmol) was dissolved in a solution of guanidine/guanidinium nitrate^36^ in MeOH/CH_2_Cl_2_ (9 : 1, 25 mL) at room temperature and after 30 min, the mixture was neutralized with AcOH and concentrated under reduced pressure. Column chromatography (SiO_2_, 4% MeOH in CH_2_Cl_2_), in which the major product eluted prior to the minor product, gave 4 (69 mg, 64%) and 5 (23 mg, 21%) as white solids. Compound 4: HRMS calcd for C_15_H_16_O_5_SNa [M + Na]^+^: 331.0616; found: 331.0615; compound 5: HRMS calcd for C_15_H_16_O_5_SNa [M + Na]^+^: 331.0616; found: 331.0618. ^13^C and ^1^H NMR data (*δ*_C_/*δ*_H_) of 4 (methanol-*d*_4_): 137.13 (C-2′), 136.46, 134.01, 130.06/8.00 (C-4′/H-4′), 129.72/7.95, 129.30/7.59, 129.16/7.92, 128.42/7.57, 128.22/8.19 (C-1′/H-1′), 122.83/7.73 (C-3′/H-3′), 96.56/4.43 (C-1/H-1), 78.37/3.38 (C-3/H-3), 71.30/3.33 (C-2/H-2), 71.15/3.95/3.25 (C-5/H-5_pro-*R*_/H-5_pro-*S*_), 70.37/3.23 (C-4/H-4). ^13^C and ^1^H NMR data (*δ*_C_/*δ*_H_) of 5 (methanol-*d*_4_): 137.66 (C-2′), 136.14, 134.32, 130.14/7.98 (C-4′/H-4′), 129.49/7.93, 129.14/7.91, 129.03/7.57, 128.38/7.55, 127.05/8.14 (C-1′/H-1′), 121.98/7.62 (C-3′/H-3′), 96.06/4.09 (C-1/H-1), 79.26/3.39 (C-3/H-3), 71.34/3.79/3.02 (C-5/H-5_pro-*R*_/H-5_pro-*S*_), 70.54/3.47 (C-4/H-4), 70.07/3.75 (C-2/H-2).

### UV and CD spectroscopy

Ultraviolet (UV) and circular dichroism (CV) spectra of the sulfoxide-containing compounds 4 and 5 were recorded on an Applied Photophysics Chirascan instrument at 20.0 °C with the wavelength range set to 260 to 190 nm.

### NMR spectroscopy for conformational analysis

NMR experiments for conformational analysis were performed on the following spectrometers: a Bruker Avance III 600 MHz spectrometer equipped with a 5 mm PFG triple resonance probe, a Bruker AVANCE 500 MHz spectrometer and a Bruker AVANCE III 700 MHz spectrometer; the latter two equipped with 5 mm TCI Z-Gradient CryoProbes. ^1^H NMR chemical shifts were referenced to external 3-trimethylsilyl-(2,2,3,3-^2^H_4_)-propanoate (TSP, *δ*_H_ 0.00) in D_2_O and ^13^C to external dioxane (*δ*_C_ 67.40) in D_2_O. The temperature was set to 37 °C with a neat deuterated methanol sample.^64^

For NMR experiments compounds 1–5 (5 mg) were dissolved in methanol-*d*_4_ (0.5 mL) and transferred to 5 mm NMR tubes. ^1^H NMR spectra were recorded with 24k data points over a 12 ppm spectral width and using 400 scans. ^13^C NMR spectra were recorded using 65k data points over a 197 ppm spectral width, zero-filled to 262k points and a 3 Hz line broadening was applied prior to Fourier transformation.

^1^H NMR chemical shifts were assigned using ^1^H,^1^H-TOCSY experiments (mixing times of 10, 40, 60 and 80 ms) and a ^1^H,^1^H-NOESY experiment (mixing time of 300 ms) with 2048 × 256 data points over a spectral width of 5 ppm. ^13^C NMR chemical shifts assignments were performed using multiplicity-edited ^1^H,^13^C-HSQC, ^1^H,^13^C-HMBC experiments. ^1^H,^13^C-HSQC experiments were recorded with 1024 × 512 data points and a spectral width of 10 and 120 ppm for ^1^H and ^13^C, respectively. The ^1^H,^13^C-HMBC experiments were carried out using 2048 × 256 data points over 10 and 120 ppm for ^1^H and ^13^C, respectively.

Selective 1D ^1^H,^1^H-NOESY experiments^42^ and selective 1DLR experiments^43,44^ were performed to study the torsional angle preferences at the glycosidic linkage in 1–5, where *ϕ* = H1–C1–X2′–C2′ and *ψ* = C1–X2′–C2′–C1′ with X = O in 1 or S in 2–5. 1D ^1^H,^1^H-NOESY experiments were acquired at 700 MHz for compounds 1–3 and at 600 MHz for compounds 4 and 5 with 8k data points, a spectral width of 8 ppm and a relaxation delay of 2 s using eight different mixing times varying from 60 to 600 ms. Selective 1DLR experiments were acquired on a 700 MHz Bruker AVANCE III spectrometer with 50k data points, 11 200 scans and a spectral width of 12 ppm. The nominal value of the long-range coupling constant was set to 8 Hz and a 160 ms selective ^13^C excitation pulse (Gauss1_90.1000) centered at the chemical shift of the C2′ resonance. Heteronuclear *J* coupling constants were extracted using the *J*-doubling method^65^ by deconvolution of the anti-phase peaks in the 1DLR spectra. The heteronuclear ^3^*J*_C2′,H1_ of compound 1 was also determined from 2D IPAP-selHSQMBC NMR experiments^66^ using a frequency-selective Gaussian shaped pulse with a duration of 40 ms, a nominal value of 5 Hz for the delay of the evolution of the long-range coupling and an acquisition time of 1.5 s. The numerical value of ^3^*J*_C2′,H1_ was extracted from IP and AP peak separations in 1D spectra extracted from rows of the 2D NMR spectra.

### Quantum mechanical calculations

Quantum mechanical geometry optimizations and calculations of NMR scalar spin–spin coupling constants were carried out as previously described.^52^

### Molecular docking simulations of the Michaelis complex

The geometries obtained for compounds 1–5 from the QM calculations carried out herein were used as initial input for the docking studies. Molecular docking with Auto-dock VINA was carried out essentially as previously described.^19^ The protein crystal structures of *Drosophila* β-1,4-galactosyltransferase 7 mutant D211N (PDB ID: 4M4K and 4LW3) were used to prepare complexes devoid of water molecules and xylose-containing acceptors but containing manganese and UDP-galactose. Furthermore, corresponding molecular models were created containing instead the catalytic base Asp211, referred to as D211. In the docking protocol used the sidechains of K176, Y177, H178, Y179, L209, E210, N211/D211 and D212 were chosen as being flexible. The top-ranked poses from the 20 docking simulations were analyzed in further detail with respect to atom–atom interactions and their geometrical arrangements.

### Biological testing

The β4GalT7 assay was carried out according to protocols described in detail previously.^4,67^

## Data availability

The datasets supporting this article have been uploaded as part of the ESI.†

## Conflicts of interest

There are no conflicts to declare.

## Supplementary Material

RA-015-D5RA00498E-s001
