# Supplementary material for: Synthesis, conformational analysis and biological activity of xylopyranosyl sulfur-containing glycosides: dependence of sulfur atom configuration
Source: RSC Adv. 2025 Jun 4;15(23):18010–20. doi: 10.1039/d5ra00498e (PMC12134886; doi:10.1039/d5ra00498e)
Supplement: RA-015-D5RA00498E-s001 [file RA-015-D5RA00498E-s001.pdf]

Supplementary Information (SI) for RSC Advances.
This journal is © The Royal Society of Chemistry 2025

Synthesis, conformational analysis and biological activity of xylopyranosyl sulfur-containing glycosides: dependence of sulfur atom configuration

Pilar Blasco[a], Jonas Ståhle[a], Karin Thorsheim[b], Axel Furevi[a], Anna Siegbahn[b], Emil Tykesson[c], Gunilla Westergren-Thorsson[c], Ulf Ellervik[b] and Göran Widmalm[a,]*

[a] Department of Organic Chemistry, Arrhenius Laboratory, Stockholm University, SE-106 91 Stockholm, Sweden

[b] Center for Analysis and Synthesis, Center for Chemistry and Chemical Engineering, Lund University, P.O. Box 124, SE-221 00 Lund, Sweden

[c] Department of Experimental Medical Science, Lund University, BMC, SE-221 00 Lund, Sweden

* Correspondence: goran.widmalm@su.se

Supporting information

Table of Contents

HRMS-table of galactosylated products S2
^{1}H and ^{13}C NMR of **7**-(*R*)s and **7**-(*S*)s mixture S3
^{1}H and ^{13}C NMR of compound **4** S4
^{1}H and ^{13}C NMR of compound **5** S5

Table S1. HRMS of galactosylated products.

Acceptor substrate	Molecular formula of galactosylation product	HSMS calcd $[M+H]^+$	HRMS found
**1**	$C_{21}H_{26}O_{10}$	439.1599	439.1598
**2**	$C_{21}H_{26}O_9S$	455.1370	455.1370
**3**	$C_{21}H_{26}O_{11}S$	487.1269	487.1270
**4** $(S)_s$	$C_{21}H_{26}O_{10}S$	471.1319	471.1320
**5** $(R)_s$	$C_{21}H_{26}O_{10}S$	471.1319	471.1320

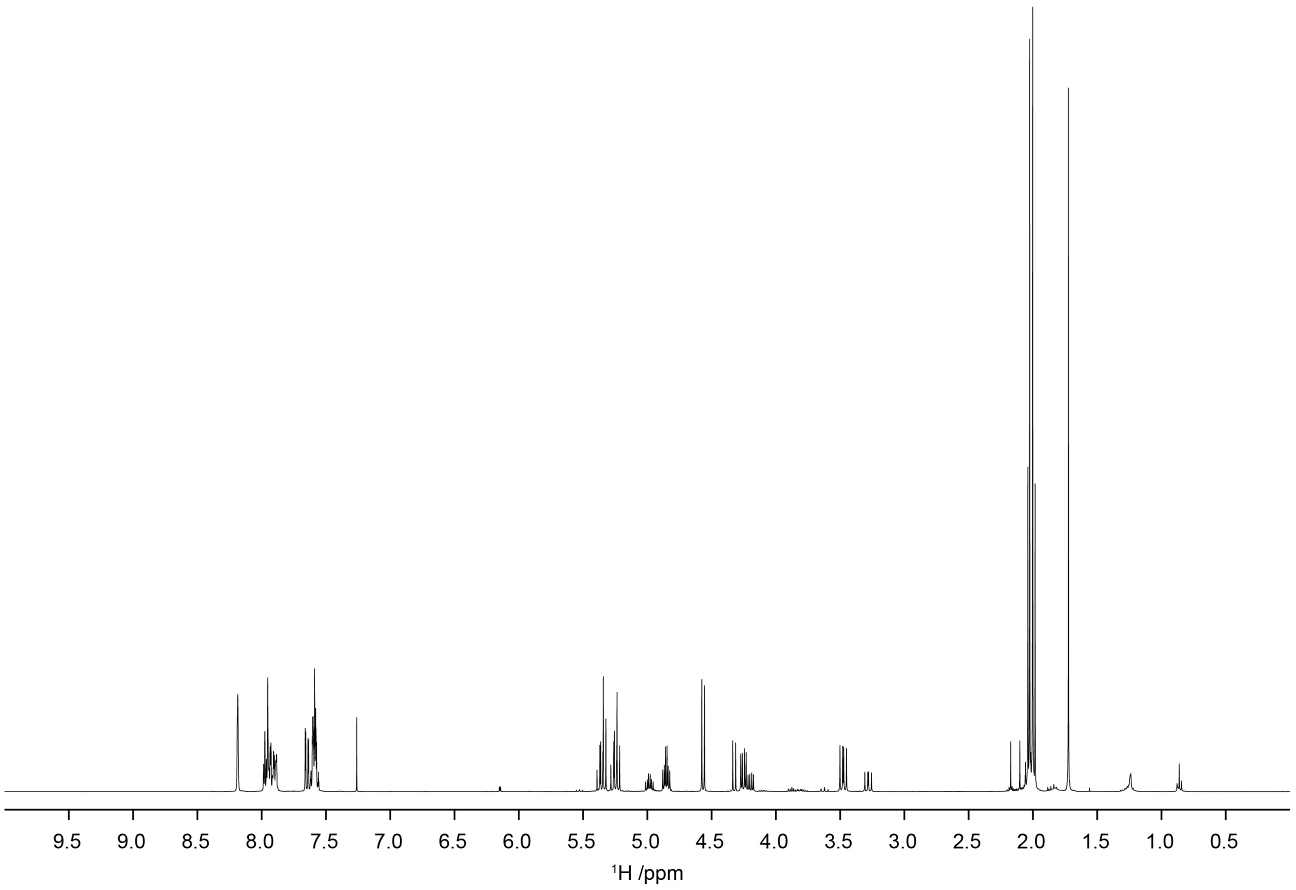

^{1}H NMR of **7**-(*R*)$_{S}$ and **7**-(*S*)$_{S}$ mixture.

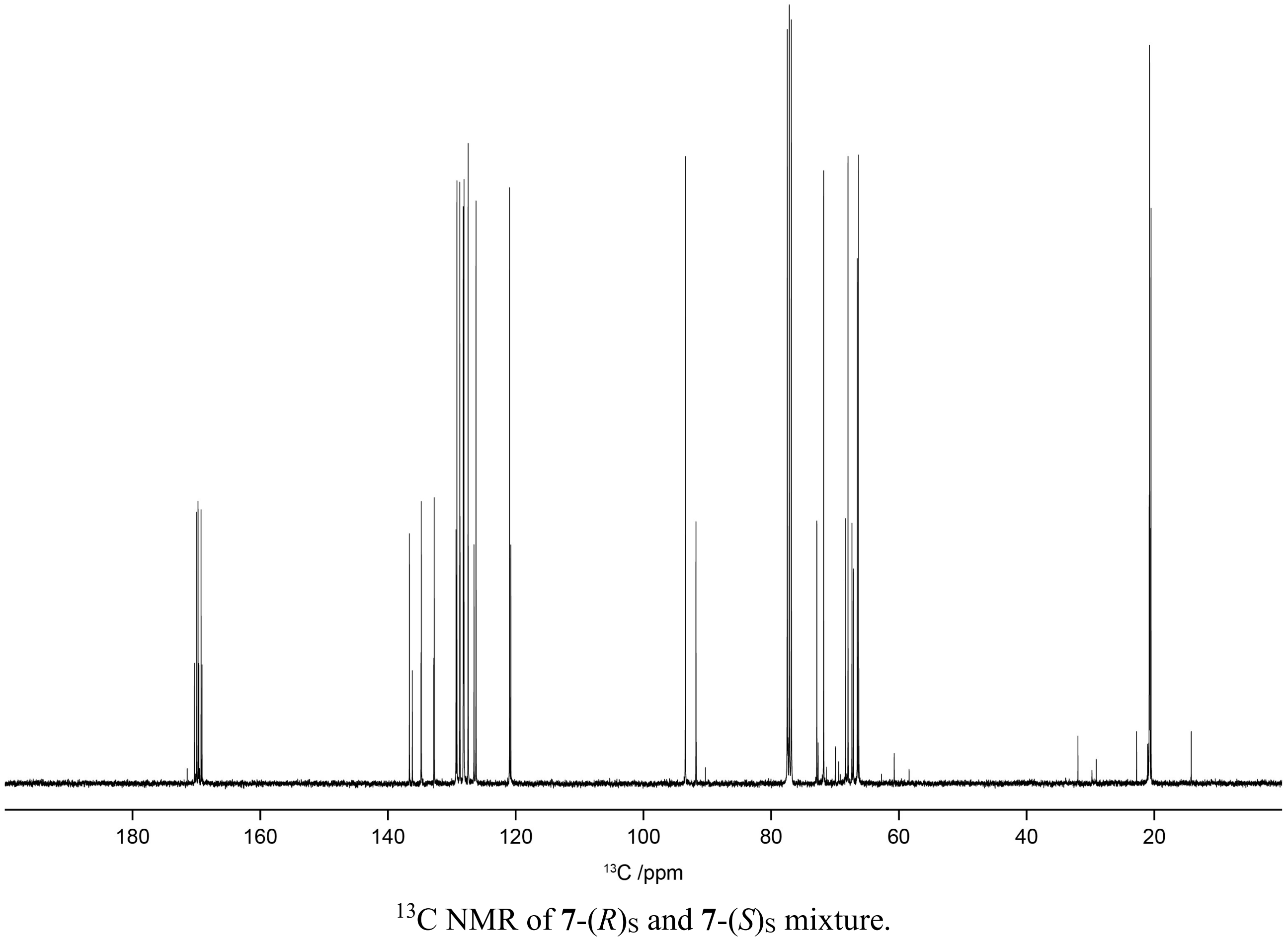

^{13}C NMR of **7**-(*R*)$_{S}$ and **7**-(*S*)$_{S}$ mixture.

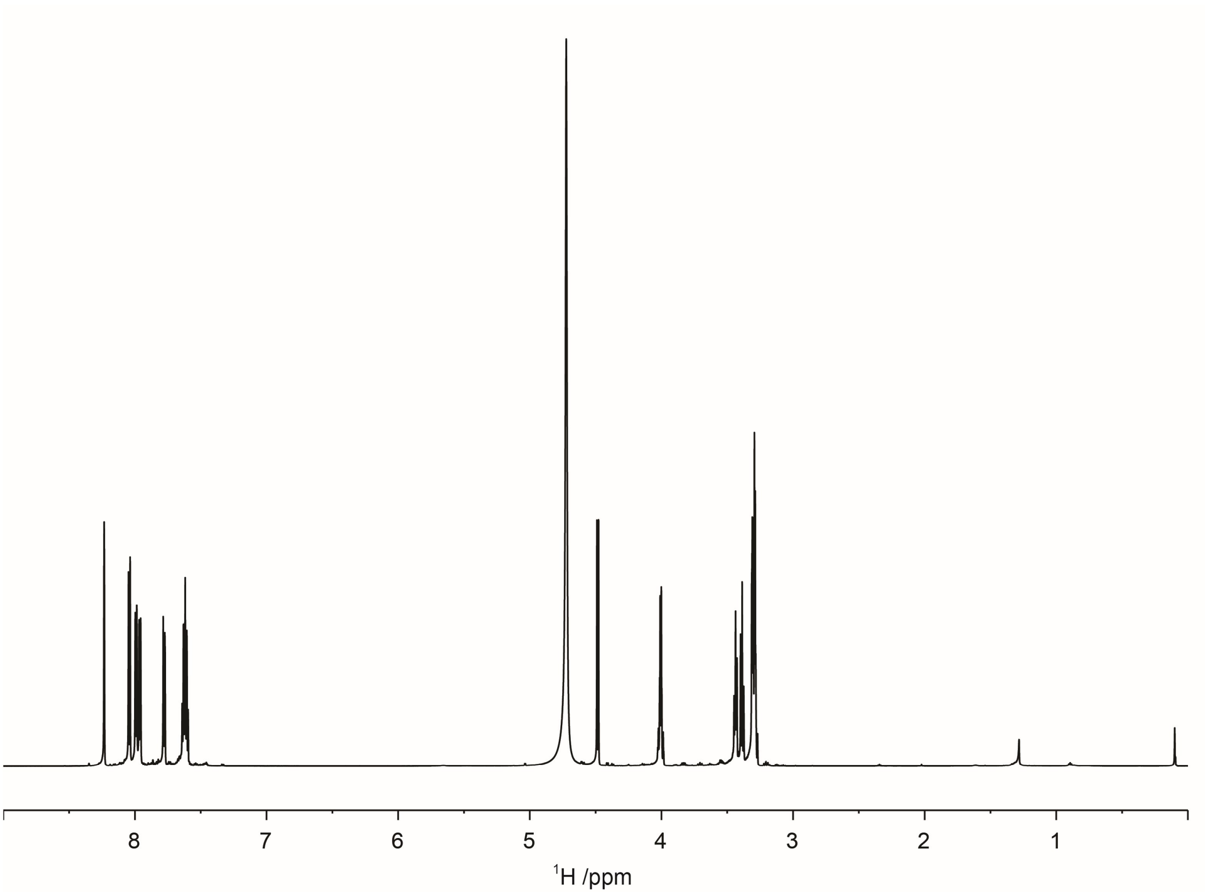

^{1}H NMR of compound **4**.

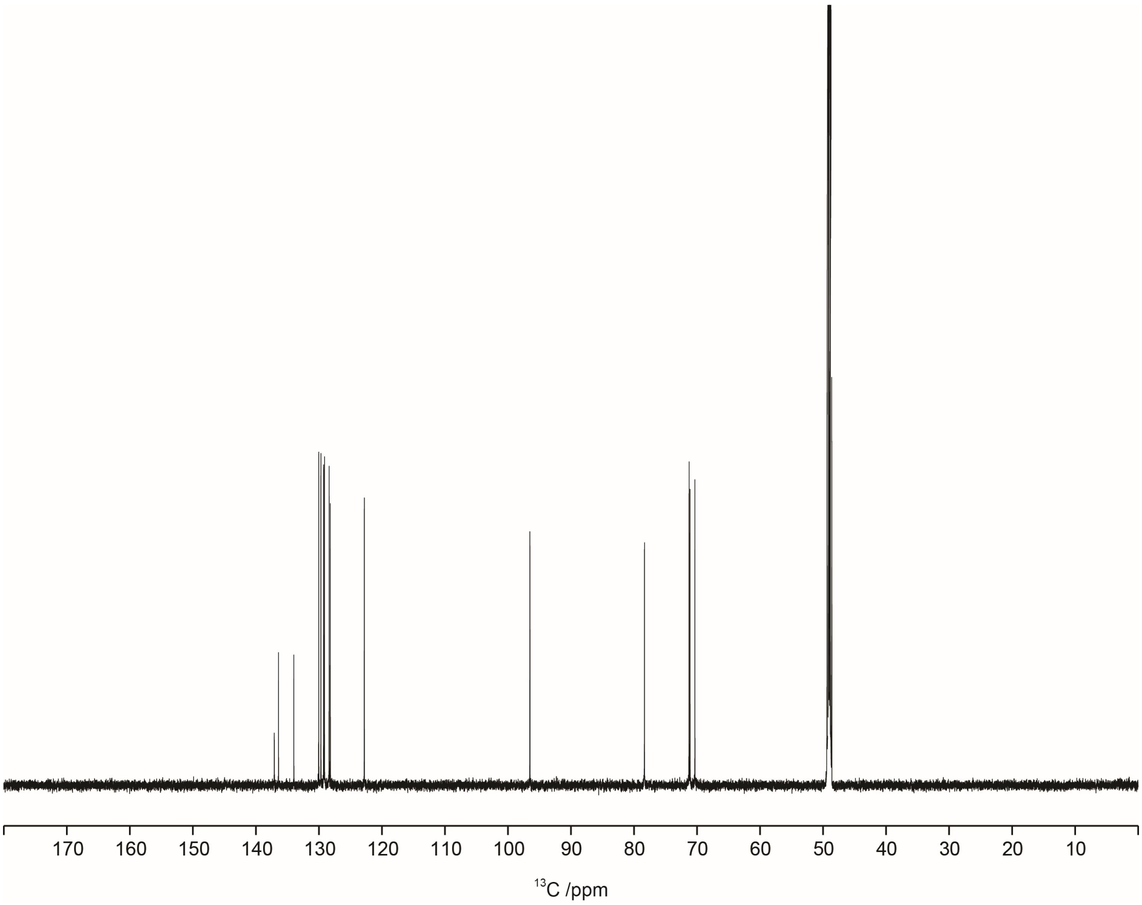

^{13}C NMR of compound **4**.

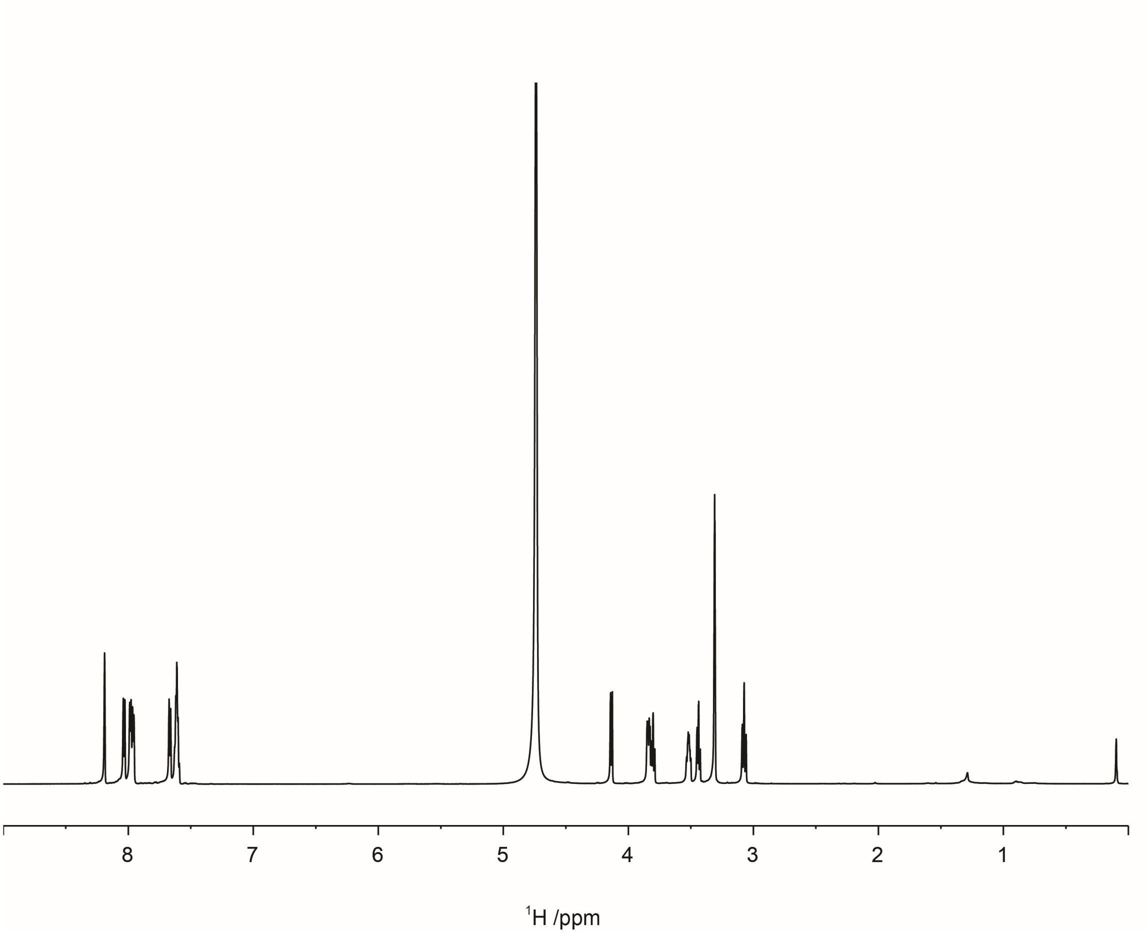

^{1}H NMR of compound **5**.

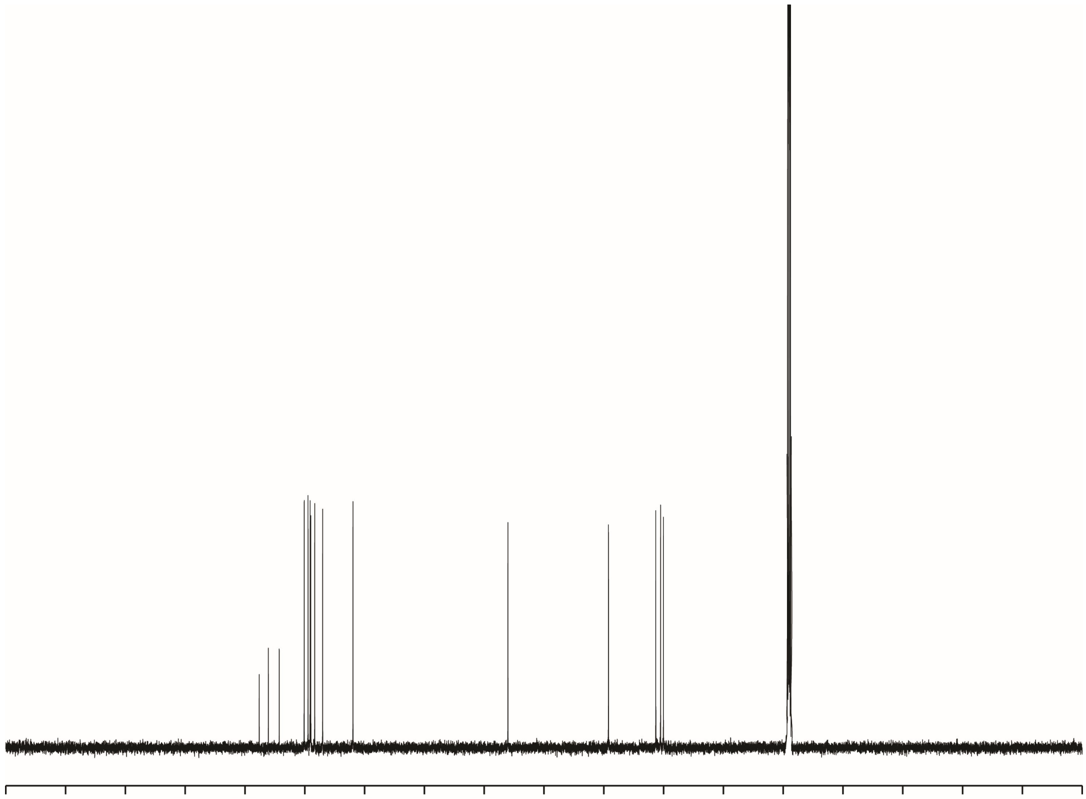

^{13}C NMR of compound **5**.
